# Changes of serum retinol-binding protein 4 associated with improved insulin resistance after laparoscopic sleeve gastrectomy in Chinese obese patients

**DOI:** 10.1186/s13098-019-0511-1

**Published:** 2020-01-14

**Authors:** Xingchun Wang, Yueye Huang, Jingyang Gao, Hang Sun, Muthukumaran Jayachandran, Shen Qu

**Affiliations:** 1 Department of Endocrinology and Metabolism, Shanghai Tenth People’s Hospital, Tongji University, School of Medicine, Shanghai, China; 2 National Metabolic Management Center, 10th Hospital, Shanghai, 200072 China

**Keywords:** Retinol binding protein 4, Insulin resistance, Laparoscopic sleeve gastrectomy, Obesity

## Abstract

**Background:**

Serum retinol-binding protein 4 (RBP4) plays a critical role in insulin resistance. The mechanism behind the impact of laparoscopic sleeve gastrectomy (LSG) on glucose metabolism is unclear. Hence, we aimed to investigate the triangle relationship between the RBP4, glucose metabolism, and LSG in patients of Chinese ethnicity.

**Methods:**

The study enrolled eighty-two obese patients. Glucose-lipid metabolic index, uric acid (UA), superoxide dismutase (SOD), free triiodothyronine (FT3), free thyroxin (FT4) and thyrotropin (TSH) were measured. RBP4 levels were detected by enzyme-link immunosorbent assay. 30 obese patients underwent LSG were studied. All these markers were measured again at a time interval of 3 and 6 months after surgery.

**Results:**

(1) Circulating RBP4 levels were positively associated with body mass index(BMI), blood glucose in 0 min (BG0), BG30, BG120, BG180, fasting inulin(FINS), fasting C peptide(FCP), homeostasis model of assessment for insulin resistance index (HOMA-IR), SOD, TSH and negatively associated with Matsuda index in obesity with a significant difference (*P *< 0.05). RBP4 levels in the patients with impaired fasting glucose (IFG), insulin resistance or hyperinsulinemia were significantly higher than the patients without IFG, insulin resistance or hyperinsulinemia (*P *= 0.035, *P *= 0.001, and *P *= 0.007). (2) LSG resulted in significantly decreased FBG, FINS, FCP and HOMA-IR at 3, 6 months after surgery (all *P *< 0.05). The RBP4 levels were significantly decreased after surgery (all *P *< 0.05) with no gender difference. (3) The change in RBP4 levels was significantly associated with the change in FINS, FCP, HOMA-IR, and HOMA-β at 6 months and the change in TSH at 3 months after surgery in males (all *P *< 0.05). The change in RBP4 levels were significantly associated with the change in FINS, FCP, HOMA-IR, HOMA-β, and TCH at 3 months after surgery in females (all *P *< 0.05).

**Conclusions:**

Overall, our results interpret the significant correlations between RBP4, glucose-lipid metabolism, oxidative stress and thyroid function in obese patients. Further, the LSG brings a decline in RBP4 levels and that may contribute partly to the improved insulin resistance in obese Chinese patients.

## Background

Lifestyle changes contribute significantly to the increased prevalence of obesity worldwide. Obesity causes type 2 diabetes and associated chronic complications [1–3]. Therefore the prevention and treatment of obesity should be given much importance. Lifestyle modifications such as dietary intervention and physical activity are regarded as first-line therapy. However, its efficacy is limited in severe or morbid obesity. The usage of weight-loss drugs are limited due to their side effects. Therefore, bariatric surgery will be a better treatment option for morbidly obese subjects or obesity with comorbidities. Previous studies have proven that bariatric surgery is effective in reducing body weight and improving glucose-lipid metabolism in obese patients [4–7].

Retinol-binding protein 4 (RBP4) is mainly released by the human liver and adipose tissue [8]. RBP4 is not just a plasma carrier of retinol but also considered as a novel adipokine that plays an important role in insulin resistance [9]. It is indicated that RBP4 concentrations are elevated in insulin-resistant mice and humans with obesity and diabetes and can be normalized by insulin-sensitizing drugs [10]. Additionally, RBP4 has a function in the fat deposition by impairing the insulin pathway in mice [11]. A study on 1033 Chinese subjects with various degrees of obesity has shown that Serum RBP4 levels were positively correlated with visceral adipose tissue (*P *< 0.001) [12]. Several other studies exhibit the interrelation between RBP4, inflammation markers and oxidative stress [8, 13].

RBP4 levels were decreased after weight loss, including bariatric surgery [14]. With context to this, a previous study showed that serum RBP4 levels are reduced after gastric banding surgery in 33 morbidly obese patients with body mass index (BMI) of 46 ± 5 kg/m^2^ [14], and the decreased RBP4 may contribute to improved insulin resistance after banding surgery with weight loss [14]. Also, a study found that the RBP4 levels start to decrease within 6 months after surgical bypass in 190 morbid obesity [15]. After banding or bypass surgery, RBP4 is decreased by about 16.6% [16], and the change in RBP4 levels are related to reductions of waist circumference (WC), waist-hip ratio (WHR) and visceral-fat diameter (all *P *< 0.0.5) [16].

To the extent of our knowledge, there is no report about the change in RBP4 levels after laparoscopic sleeve gastrectomy (LSG) in obese patients of Chinese ethnicity. We hypothesis that change in RBP4 may associate with the improvement of glucose metabolism after LSG. Hence, this study was designed to investigate the association between RBP4 and glucose-lipid metabolic markers in patients of Chinese ethnicity and change in RPB4 levels after LSG.

## Materials and methods

### Subjects

An observational study of 34 men and 48 women subjects with obesity were enrolled from the Endocrine and Metabolism Department of Shanghai Tenth People’s Hospital. The obesity was defined by BMI over 30 kg/m^2^. 10 healthy persons with normal BMI (average BMI 20.22 ± 1.15 kg/m^2^) were included as controls. Among obese patients, 30 subjects (males:female = 10:20) underwent LSG. Inclusion criteria: BMI ranged from 31.16 to 52 kg/m^2^ and age ranged from 18 to 60 years old which meets the recommended cutoff for bariatric surgery in Asians. Exclusion criteria: (1) secondary cause of obesity: hypothalamic obesity, Cushing syndrome, and hypophysis dysfunction, etc., (2) pregnancy or lactation, (3) taking antipsychotic medication, (4) presence of severe complications of obesity and diabetes, (5) Gastrointestinal diseases that contraindications for laparoscopic surgery, such as intra-abdominal infection, adhesions, etc., (6) Severe organic and systemic diseases intolerant of surgery (e.g. malignant tumor, severe heart, liver and kidney dysfunction, mental illness, autoimmune disease, acute or chronic inflammation, etc.). These subjects were examined before the LSG and followed up at 3, 6 months after surgery. For all patients in this cohort, this was the primary bariatric procedure and none have required an additional bariatric operation. The operation was performed by a professional surgeon under standard procedures, and under general anesthesia induced with a tracheal cannula. After CO_2_ pneumoperitoneum was established, a laparoscope was used to explore the abdominal cavity. Four other trocars were inserted through incisions alongside the bilateral rectus abdominis muscles, the right side of the midline, and below the costal margin. A LigaSure Atlas (Covidien, Inc.) was used to take down the inner side of the hemal arch from the greater curvature to the fundus of the stomach entirely. A stent was planted in the lesser curvature, and an Echelon (Ethicon Endo-Surgery, Inc.) was used to cut and close the stomach from the pylorus ring to the fundus of the stomach several times to make the stomach like a tube. A Stratafix (Ethicon Endo-Surgery, Inc.) was used to make a continuous suture in the greater curvature of the stomach. Then, the incision was checked to avoid bleeding, and part of the stomach from navel incision was taken out. Negative pressure drainage was applied in the splenic recess and led to the right incision. Informed consent was signed and obtained by all individual participants included in this study. This study was approved by the Shanghai Tenth People’s ethical committee.

### Anthropometric measurements

Anthropometric data including body weight and height were measured without shoes and with light clothing by professional staff. We calculated the BMI by weight in kilograms divided by height in meters squared. WC and hip circumference (HC) were also measured. WC was measured at the midway between the lower rib margin and the iliac crest. HC was measured at the top point around the buttocks. The WHR was calculated as HC divided by WC.

### Laboratory tests

Venous blood samples were adopted from all the subjects at least fasting for 8 h at baseline and 3, 6 months after LSG in the subjects under LSG. The serum was centrifuged immediately and stored at − 80 °C. Lipid metabolic markers [total cholesterol(TCH), triglyceride (TG), high density lipoprotein cholesterol (HDL-C), low density lipoprotein cholesterol (LDL-C), free fatty acid (FFA)], uric acid (UA) and superoxide dismutase (SOD) were measured by Roche Cobas c701 fully automatic biochemical analyzer. Free triiodothyronine (FT3), free thyroxin (FT4) and thyroid-stimulating hormone (TSH) were measured by ADVIA Centaur XP Immunoassay System. A 75 g-oral glucose tolerance test (OGTT) was performed to estimate glucose metabolism and insulin clearance according to the methods demonstrated by World Health Organization [17]. The glucose, insulin and C-peptide levels at 0, 30, 60, 120 and 180 min were measured. Glucose was measured by by Roche Cobas c701 fully automatic biochemical analyzer, insulin and C-peptide were measured by Roche Cobas e 601 analyzer.

The HOMA of insulin resistance (HOMA-IR) was calculated to assess insulin resistance by the following formula: fasting serum insulin concentration (uIU/mL) × fasting blood glucose concentration (mmol/L)/22.5 [18]. HOMA-β was calculated to evaluate beta-cell function: HOMA-β = 20 × fasting insulin/(fasting glucose − 3.5) [18]. Insulin sensitivity was evaluated by the Matsuda index. Matsuda index was calculated as the following formula: ${\text{Matsuda index}}\, = \, 1000/\sqrt {Glu0 \times INS0 \times MeanGlu \times MeanIns}$ [19]. Serum retinol-binding protein 4 was detected by enzyme-link immunosorbent assay (R&D Systems, Catalog Number DRB400).

### Definition of glucose-lipid metabolic disorders

Impaired fasting glucose (IFG) was defined by fasting glucose levels ≥ 5.6 mmol/L [20]. Hyperinsulinemia and insulin resistance (IR) were defined by FINS concentrations ≥ 15 mU/L and HOMA-IR ≥ 2.5 respectively [21]. Hypertriglyceridemia, hypercholesterolemia, low HDL-C, and high LDL-C were defined by fasting plasma TG ≥ 1.7 mmol/L, TCH ≥ 5.18 mmol/L, LDL-C ≥ 3.37 mmol/L and HDL-C < 1.04 mmol/L respectively.

### Statistical analysis

SPSS20.0 statistical software was used to analyze the data. Descriptive statistics were expressed as mean ± standard deviation (X ± SD). Count data were expressed as number (n). An independent sample-test was used to compare the data between two independent groups. Statistical significance before and after surgery was evaluated using the paired two-tailed t-test. Nonparametric tests were used for non-normal distributed data. Pearson’s and Spearman’s correlation coefficient was used for correlations between RBP4 levels and other markers. The difference was considered significant at a *P* value of less than 0.05.

## Results

### Baseline characteristics

**Fig. 1:**
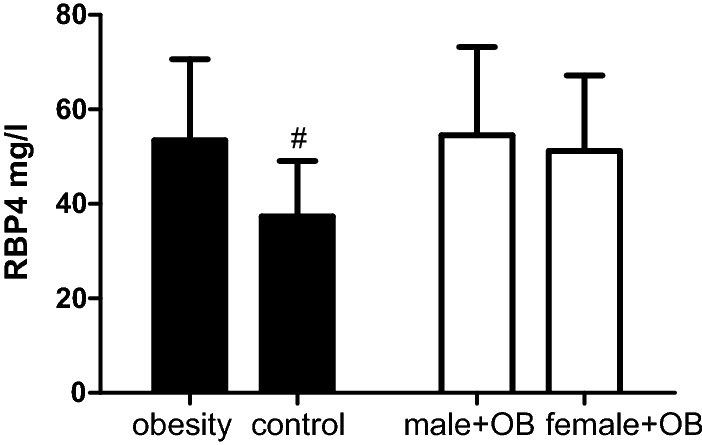
RBP4 levels among groups. *RBP4* retinol-binding protein 4, *OB* obesity; compared to obesity, ^#^*P *< 0.001

There were a total of 82 obese patients enrolled (males:females = 34:48) with average BMI 35.20 ± 6.69 kg/m^2^. 10 healthy persons aged 32.08 ± 9.84 years old (males:females = 4:6) with average BMI 20.22 ± 1.15 kg/m^2^ were enrolled as a control group. RBP4 levels were significantly higher in obese patients than healthy weight controls (53.53 ± 17.12 mg/L vs. 37.39 ± 11.70 mg/L, *P *< 0.001) as presented in Fig. 1. RBP4 levels between genders show no significant difference in obese patients (54.59 ± 18.66 vs. 51.23 ± 15.94 mg/L, *P *= 0.416). Additionally, the average BMI of 30 obese subjects (males:female = 10:20) who underwent LSG was 38.97 ± 4.83 kg/m^2^ and the average age of them was 33.16 ± 11.03 years old. Markers of glucose-lipid metabolism of the 30 obesity subjects were presented in Table 2.

### Correlations of serum RBP4 levels with other parameters

**Fig. 2:**
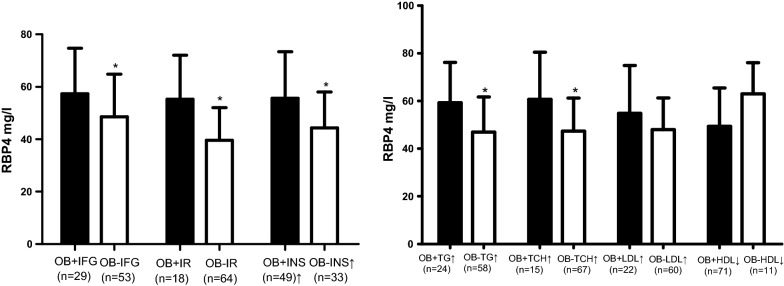
RBP4 levels in all obesity with abnormal metabolism. *RBP4* retinol-binding protein 4, *OB* obesity, *IFG* impaired fasting glucose, *IR* insulin resistance, *INS* insulin, *TG* triglyceride, *TCH* total cholesterol, *LDL* low-density lipoprotein cholesterol, *HDL* high-density lipoprotein cholesterol; *P < 0.05

RBP4 levels were significantly and positively associated with BMI, BG0, BG30, BG120, BG180, INS0, CP0, HOMA-IR, SOD, TSH and negatively associated with Matsuda index in all obesity (all *P *< 0.05). RBP4 levels were significantly and positively associated with UA, BG0, BG30, BG120, BG180, INS0, CP0, HOMA-IR, HGB, SOD, TCH and TG in females with obesity (all *P *< 0.05). RBP4 levels were only significantly and positively associated with HOMA-IR, SOD and negatively with the Matsuda index in males with obesity (all *P *< 0.05). All the results were showed in Table 1. Among the obese subjects, 41.1% had IFG, 77.7% had insulin resistance and 68.6% had hyperinsulinemia. RBP4 levels of the patients with IFG, insulin resistance or hyperinsulinemia were significantly higher than the patients without IFG, insulin resistance or hyperinsulinemia (57.39 ± 17.34 mg/L vs. 48.52 ± 16.29 mg/L, *P *= 0.035; 55.18 ± 16.90 mg/L vs. 39.57 ± 12.45 mg/L, *P *= 0.001; 55.58 ± 17.81 mg/L vs. 44.33 ± 13.67 mg/L, *P *= 0.007). Additionally, RBP4 levels of the patients with hypertriglyceridemia or hypercholesterolemia were significantly higher than patients with normal TG or TCH (59.36 ± 16.82 mg/L vs. 46.92 ± 14.76 mg/L, *P *= 0.029, and 60.66 ± 19.74 mg/L vs. 47.36 ± 13.88 mg/L, *P *= 0.028). RBP4 levels of the patients with higher LDL-C or lower HDL-C were slighter higher than corresponding control groups (54.85 ± 20.04 mg/L vs. 47.96 ± 13.36 mg/L, *P *= 0.205 and 49.38 ± 16.10 mg/L vs. 63.00 ± 13.11 mg/L, *P *= 0.164). All the data were presented in Fig. 2.

**Table 1:** Correlation of RBP4 with metabolic parameters

Parameter N = 82	All obesity		Males		Females	
	r	*P*	r	*P*	r	*P*
BMI	0.376	0.001	0.207	0.281	0.208	0.216
WC	0.301	0.136	0.253	0.428	− 0.86	0.770
HC	0.185	0.366	0.308	0.330	− 0.348	0.223
WHR	0.322	0.108	− 0.144	0.302	0.403	0.153
BG0 min	0.351	0.003	0.356	0.058	0.346	0.031
BG30 min	0.303	0.023	0.041	0.838	0.408	0.028
BG60 min	0.241	0.073	0.009	0.963	0.300	0.113
BG120 min	0.289	0.024	0.173	0.369	0.401	0.023
BG180 min	0.295	0.027	0.248	0.212	0.378	0.043
INS0 min	0.349	0.004	0.249	0.184	0.425	0.011
INS30 min	0.078	0.565	0.201	0.316	− 0.214	0.265
INS60 min	0.033	0.810	0.305	0.122	− 0.253	0.185
INS120 min	0.109	0.403	0.116	0.548	0.108	0.557
INS180 min	0.260	0.053	0.244	0.220	0.273	0.152
CP0 min	0.283	0.022	0.243	0.187	0.417	0.014
CP30 min	0.030	0.826	0.071	0.724	− 0.108	0.583
CP60 min	− 0.161	0.240	0.040	0.844	− 0.286	0.140
CP120 min	− 0.089	0.503	0.034	0.861	0.084	0.658
CP180 min	0.195	0.153	0.119	0.554	0.267	0.169
HOMA-IR	0.412	0.001	0.420	0.026	0.418	0.012
HOMA-β	− 0.117	0.362	0.124	0.530	0.063	0.721
Matsuda index	− 0.399	0.003	− 0.480	0.015	− 0.307	0.106
HGB	0.263	0.065	0.085	0.708	0.408	0.031
TCH	0.175	0.143	0.146	0.427	0.492	0.001
TG	0.079	0.514	0.028	0.878	0.444	0.005
HDL-C	− 0.017	0.889	0.068	0.710	0.111	0.500
LDL-C	0.118	0.327	0.163	0.371	0.221	0.177
FFA	0.117	0.336	0.101	0.581	0.175	0.292
UA	0.142	0.238	− 0.046	0.806	0.371	0.019
SOD	0.486	< 0.001	0.603	0.001	0.381	0.041
FT3	0.205	0.094	0.321	0.073	0.127	0.461
FT4	− 0.086	0.480	− 0.159	0.384	− 0.033	0.844
TSH	0.274	0.023	0.324	0.071	0.099	0.560

*BMI* body mass index, *WC* waist circumference, *HC* hip circumference, *WHR* waist to hip ratio, *BG* blood glucose, *INS* insulin, *CP* C peptide, *HOMA-IR* homeostasis model of assessment for insulin resistance index, *HGB* glycosylated hemoglobin, *TG* triglyceride, *TCH* total cholesterol, *LDL-C* low density lipoprotein cholesterol, *HDL-C* high density lipoprotein cholesterol, *FFA* free fatty acid, *UA* uric acid, *SOD* superoxide dismutase, *FT3: FT4* free thyroxine, *TSH* thyroid stimulating hormone

### Improved metabolism and decreased serum RBP4 levels after surgery

**Fig. 3:**
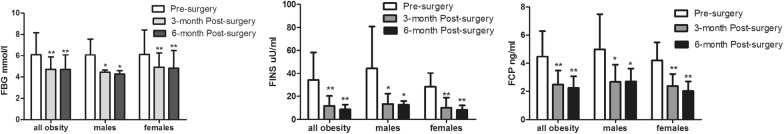
Change in glucose metabolism. *FBG* fasting blood glucose, *FINS* fasting insulin, *FCP* fasting C-peptide; *P < 0.05; **P < 0.01

**Fig. 4:**
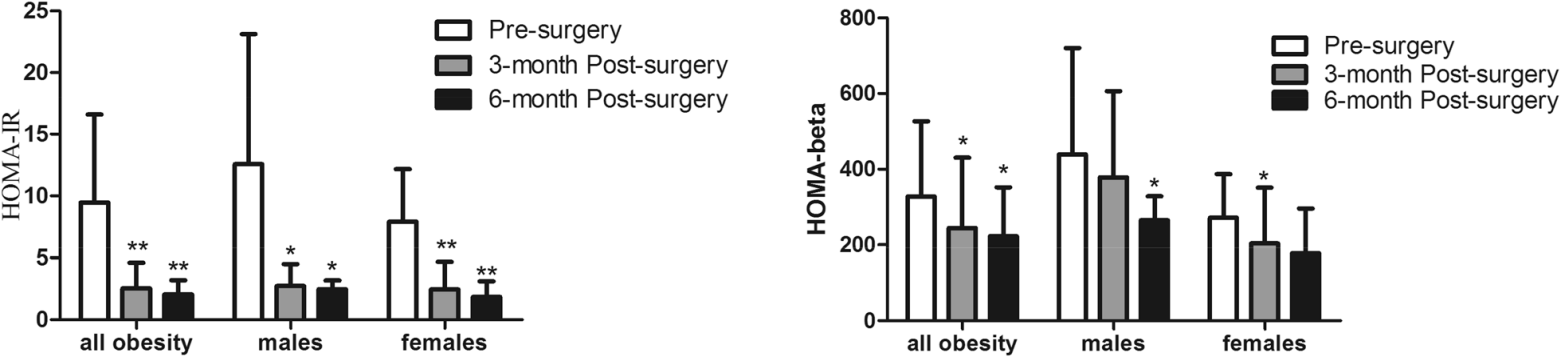
Change in insulin resistance. *HOMA-IR* homeostasis model of assessment for insulin resistance index; *P < 0.05; **P < 0.01

**Fig. 5:**
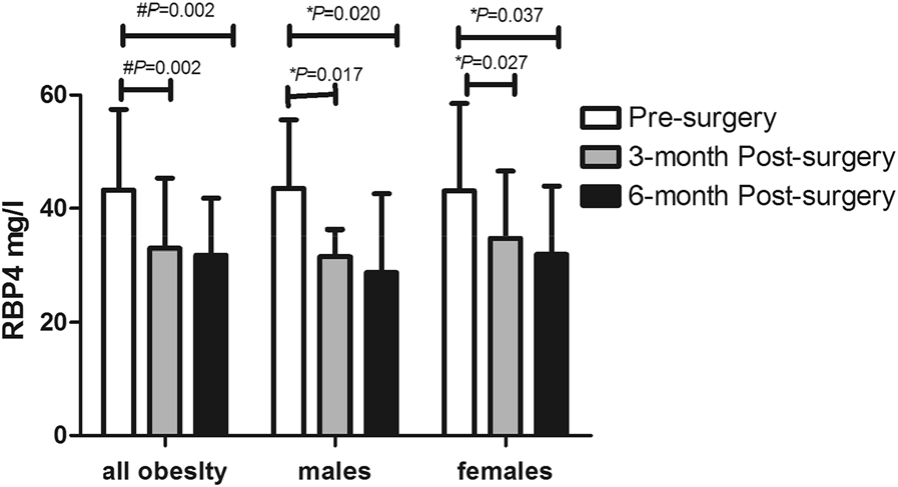
Change in RBP4 levels. *RBP4* retinol-binding protein 4; *P < 0.05; ^#^*P *< 0.01

Weight, BMI, WC, HC and, TG were significantly decreased in males and females at 3, 6 months after surgery (all *P *< 0.05). TG at 3 and 6 months, UA at 6 months, SOD at 3 months after surgery were significantly decreased and HDL-C at 3, 6 months after surgery were significantly increased in females (all *P *< 0.05). SOD and HDL-C were significantly increased at 6 months after surgery in males (all *P *< 0.05). All the results were showed in Table 2. LSG led to significantly decreased fasting glucose, FINS, and FCP at 3, 6 months after surgery (all *P *< 0.05) as shown in Fig. 3. HOMA-IR at 3, 6 months in both genders, HOMA-β at 3 months in females and at 6 months in males were significantly decreased after surgery (all *P *< 0.05) as presented in Fig. 4. Additionally, RBP4 levels were significantly decreased in all subjects after surgery with a significant difference (all *P *= 0.002). RBP4 levels were significantly decreased from 43.50 ± 12.11 to 31.6 ± 4.74 mg/L at 3 months, and to 28.83 ± 13.74 mg/L at 6 months after surgery in males (*P *= 0.020 and *P *= 0.017). RBP4 levels were significantly decreased from 43.07 ± 15.48 to 34.79 ± 11.76 mg/L at 3 months, and to 32.00 ± 11.94 mg/L at 6 months after surgery in females (*P *= 0.037 and *P *= 0.027). It need be mentioned that RBP4 levels were slightly decreased from 3 months to 6 months after surgery without statistic difference in females, males and all subjects with obesity (all *P *> 0.05). All these results were showed in Fig. 5. RBP4 levels decreased by 16.7% and 19.6% in all obesity, 34.1% and 30.8% in males, 9.2% and 18.9% in females at 3 and 6 months after surgery respectively.

**Table 2:** Metabolic markers after surgery

Parameter N = 30	All obesity			Males			Females		
	Pre-surgery	3 M Post surgery	6 M Post surgery	Pre-surgery	3 M Post surgery	6 M Post surgery	Pre-surgery	3 M Post surgery	6 M Post surgery
Weight,kg	113.72 ± 38.40	85.74 ± 17.40^#^	78.08 ± 14.07^#^	141.32 ± 54.41	101.29 ± 16.99*	88.05 ± 8.94^#^	99.19 ± 12.96	77.56 ± 11.03^#^	73.43 ± 13.77^#^
BMI, kg/m^2^	38.63 ± 4.60	30.46 ± 4.40^#^	27.90 ± 4.01^#^	40.44 ± 5.39	32.33 ± 5.34^#^	28.12 ± 2.96^#^	37.68 ± 3.96	29.46 ± 3.58^#^	27.80 ± 4.52^#^
WC, cm	117.34 ± 10.97	98.94 ± 10.21^#^	92.47 ± 10.77^#^	126.14 ± 7.42	104.07 ± 10.28^#^	95.85 ± 8.76^#^	113.91 ± 10.30	96.94 ± 9.74^#^	90.90 ± 11.53^#^
HC, cm	120.98 ± 6.78	106.08 ± 7.73^#^	101.68 ± 8.65^#^	122.71 ± 5.90	108.57 ± 5.50^#^	102.21 ± 5.64^#^	120.30 ± 7.14	105.11 ± 8.37^#^	101.43 ± 9.92^#^
TCH, mmol/L	4.71 ± 1.23	4.47 ± 0.84	4.41 ± 0.87	4.60 ± 1.58	4.08 ± 1.09	4.14 ± 1.15	4.78 ± 1.04	4.60 ± 0.68	4.62 ± 0.65
TG, mmol/L	2.07 ± 1.87	1.17 ± 0.36*	0.96 ± 0.43^#^	1.99 ± 1.92	1.01 ± 0.25	0.84 ± 0.28	2.11 ± 1.90	1.26 ± 0.40*	1.02 ± 0.48*
HDL-C, mmol/L	1.01 ± 0.19	1.06 ± 0.21*	1.23 ± 0.22^#^	0.98 ± 0.16	1.10 ± 0.20	1.20 ± 0.19*	1.03 ± 0.21	1.04 ± 0.22*	1.24 ± 0.23^#^
LDL-C, mmol/L	2.71 ± 0.85	2.79 ± 0.71	2.83 ± 0.74	2.59 ± 0.94	2.59 ± 0.93	2.61 ± 0.92	2.77 ± 0.83	2.91 ± 0.55	2.94 ± 0.65
UA, umol/L	408.52 ± 91.90	393.54 ± 101.04	366.42 ± 72.78*	448.56 ± 89.91	475.56 ± 85.28	412.47 ± 82.83	383.50 ± 86.55	342.28 ± 73.24	337.12 ± 49.59*
SOD, U/mL	167.13 ± 32.73	164.00 ± 28.90	173.87 ± 19.40	160.44 ± 17.18	181.00 ± 29.43	191.80 ± 22.87*	171.76 ± 40.26	152.23 ± 22.75*	165.72 ± 11.03
TSH, mU/L	2.87 ± 1.60	2.68 ± 1.65	2.41 ± 1.81	3.17 ± 1.82	2.63 ± 1.94	3.42 ± 2.30	2.70 ± 1.49	2.27 ± 1.78	2.27 ± 1.09

*BMI* body mass index, *WC* waist circumference, *HC* hip circumference, *TG* triglyceride, *TCH* total cholesterol, *LDL-C* low density lipoprotein cholesterol, *HDL-C* high density lipoprotein cholesterol, *FFA* free fatty acid, *UA* uric acid, *SOD* superoxide dismutase, *TSH* thyroid stimulating hormone

^#^*P *< 0.001, * *P *< 0.05

### Change in RBP4 levels associated with a change in metabolic markers after surgery

In males, the change in serum RBP4 levels were significantly associated with a change in FINS, FCP, HOMA-IR and HOMA-β at 6 months and were associated with a change in TSH at 3 months after surgery (all *P *< 0.05). In females, the change in serum RBP4 levels were significantly related to change in FINS, FCP, HOMA-, HOMA-β and TCH at 3 months after surgery (all *P *< 0.05). The change in serum RBP4 levels were not significantly associated with the change in lipid profile while were significantly associated with FCP at 6 months in all obesity (all *P *> 0.05). All results are presented in Table 3.

**Table 3:** Change in RBP4 levels and metabolic markers

Parameter (N = 30)	All obesity		Males		Females	
	r	*P*	r	*P*	r	*P*
3M-del weight	0.388	0.091	0.371	0.468	0.315	0.273
6M-del weight	0.199	0.495	− 0.359	0.553	0.326	0.391
3M-del BMI	0.208	0.379	− 0.143	0.787	0.185	0.527
6M-del BMI	0.093	0.752	− 0.667	0.219	0.310	0.417
3M-del WC	− 0.006	0.983	0.500	0.667	− 0.205	0.502
6M-del WC	− 0.322	0.262	− 0.308	0.614	− 0.444	0.232
3M-del HC	− 0.030	0.914	− 0.500	0.667	− 0.199	0.515
6M-del HC	− 0.344	0.228	− 0.564	0.332	− 0.206	0.595
3M-del TCH	0.169	0.477	− 0.200	0.704	0.537	0.048
6M-del TCH	0.294	0.330	− 0.667	0.219	0.502	0.168
3M-del TG	0.122	0.609	− 0.257	0.623	0.295	0.306
6M-del TG	0.184	0.530	0.103	0.870	0.261	0.498
3M-del HDL-C	0.008	0.972	0.314	0.544	0.180	0.539
6M-del HDL-C	− 0.075	0.798	− 0.103	0.870	− 0.192	0.620
3M-del LDL-C	− 0.061	0.798	− 0.371	0.468	0.022	0.940
6M-del LDL-C	0.155	0.597	− 0.154	0.805	0.226	0.559
3M-del FBG	0.046	0.850	0.000	1.000	0.152	0.605
6M-del FBG	0.062	0.833	− 0.103	0.870	0.308	0.420
3M-del FINS	0.377	0.101	− 0.371	0.468	0.673	0.008
6M-del FINS	0.135	0.646	− 0.975	0.005	0.586	0.097
3M-del FCP	0.368	0.110	− 0.371	0.468	0.642	0.013
6M-del FCP	0.498	0.029	− 0.975	0.005	0.510	0.160
3M-del HOMA-IR	0.394	0.096	− 0.800	0.104	0.673	0.008
6M-del HOMA-IR	0.084	0.775	− 0.975	0.005	0.586	0.097
3M-del HOMA-β	0.278	0.248	0.100	0.873	0.649	0.012
6M-del HOMA-β	0.124	0.673	− 0.975	0.005	0.653	0.057
3M-del TSH	0.242	0.303	− 0.841	0.036	− 0.240	0.568
6M-del TSH	− 0.326	0.276	− 0.667	0.219	0.506	0.065

*BMI* body mass index, *WC* waist circumference, *HC* hip circumference, *FINS* fasting insulin, *FCP* fasting C peptide, *HOMA-IR* homeostasis model of assessment for insulin resistance index, *TG* triglyceride, *TCH* total cholesterol, *LDL-C* low density lipoprotein cholesterol, *HDL-C* high density lipoprotein cholesterol, *TSH* thyroid stimulating hormone

## Discussion

As a metabolic risk factor in obesity, RBP4 has been reported to be associated with insulin resistance and adipose accumulation. RBP4 levels are elevated in obesity and are positively associated with BMI and WHR [22–27]. In our study, RBP4 levels were also significantly higher in obese patients than healthy controls with normal BMI and RBP4 levels were also significantly associated with BMI in obesity. Additionally, RBP4 levels may decrease after weight loss. A study proved that RBP4 levels were reduced 6 months after gastric banding in morbidly obese subjects [14]. Decreased RBP4 is associated with changes in BMI, HOMA-IR and TCH (all *P *< 0.05) [14]. Another study compared the SG to roux-en-y gastric bypass(GBP) in subjects with diabetes, the results showed that RBP4 was significantly decreased as early as 3 days after GBP [28]. By taking these facts into consideration, in this study, we have investigated the relationship between RBP4 and the other metabolic markers and the change of its levels after LSG. We demonstrated a marked decrease in serum RBP4 levels at 3 and 6 months after LSG. To our knowledge, this study is the first study to assess the change in RBP4 serum levels after LSG.

RBP4 was mostly secreted by the human liver and adipose tissue as an adipokine [8]. RBP4 contributes to the pathogenesis of type 2 diabetes. A study by Kahn BB has first documented the importance of RBP4 in insulin resistance [10]. Adipose-specific GLUT4 knockout [adipose-Glut4 (−/−)] mice show insulin resistance is related to increased RBP4 expression [10]. Additionally, the overexpression of RBP4 or injection of recombinant RBP4 in wild-type mice leads to the development of insulin resistance [10]. In this study, RBP4 levels were significantly and positively associated with BG0, BG30, BG120, BG180, INS0, CP0, HOMA-IR and HGB in females with obesity, and also significantly and positively associated with HOMA-IR and negatively with Matsuda index in males with obesity. Additionally, RBP4 levels of the patients with IFG, insulin resistance or hyperinsulinemia were significantly higher than the patients without IFG, insulin resistance or hyperinsulinemia. These results are consistent with previous studies that show RBP4 is significantly related to glucose metabolism in the human [10].

In addition to the influence of RBP4 on glucose metabolism, it also has a specific role in lipid metabolism. A previous study proved that elevated serum RBP4 is associated with increased serum TG levels and decreased HDL-C levels [27]. In this similar pattern, our study results show RBP4 levels were significantly and positively associated with TCH and TG in females with obesity but not in males. Additionally, RBP4 levels of patients with hypertriglyceridemia or hypercholesterolemia were significantly higher than patients with normal TG or TCH in females. Therefore, we can conclude that RBP4 interference with lipid metabolism is gender-dependent.

As a novel adipokine secreted by adipocyte, RBP4 is significantly higher in obese subjects than adults with normal weight [29, 30]. RBP4 levels are also associated with oxidative stress and inflammatory markers [13, 31]. There exists a positive association of RBP4 levels with oxidative stress markers such as SOD in males and females with obesity in this study. An interesting study demonstrates that plasma RBP4 levels are significantly associated with UA in the Chinese population [32]. In conclusion, RBP4 levels were found significant with serum UA levels in females with obesity.

TSH levels present a positive association with BMI and are increased in obese patients when compared to the control group with normal weight [33]. Obese patients with the complication of mild thyroid hormone deficiency exert more serious metabolic disturbance [34]. However, few studies involved studying the association of RBP4 levels with thyroid function in obesity. A previous study showed that RBP4 levels were positively associated with TSH in postmenopausal women [35]. RBP4 levels are higher in patients with clinical hypothyroidism than controls [36]. In this study, RBP4 levels were significantly and positively associated with TSH in obesity and the change in serum RBP4 levels were associated with change in TSH in 3rd month after LSG in males. Hence, we suggest that RBP4 may play a role in thyroid dysfunction in obesity.

Metabolic surgery is more and more popular worldwide for reducing body weight and improving metabolism. Insulin resistance is significantly improved earlier than weight loss after surgery [37]. In our study, body weight was significantly reduced and the metabolism was significantly improved at 3, 6 months after LSG. Glucose metabolism improved especially accompanied by decreased fasting glucose, FINS, FCP, HOMA-IR and HOMA-β after surgery. However, the mechanism by which bariatric surgery improves glucose metabolism is unclear. Change of hormone secretion in the gastrointestinal tract may be one of those mechanisms [38, 39]. RBP4 plays an important role in insulin resistance, and the improved glucose metabolism after LSG may be related to RBP4. A previous study illustrated the changes in RBP4 levels after gastric banding in morbidly obese subjects [14]. Another study also confirmed the change in RBP4 levels after GBP as early as 3 days [28]. While, they did not found change in RBP4 levels at 3 days after SG [28]. Therefore, we investigated the change in RBP4 levels after LSG with follow up time of 6 months. The results of our study showed that RBP4 levels were significantly decreased at 3, 6 months after surgery in males and females. Therefore, there exists change in RBP4 levels with long term after SG. RBP4 levels were closely associated with insulin resistance. Additionally, we found that the change in serum RBP4 levels were associated with change in FINS, FCP, HOMA-IR and HOMA-β at 6th month after LSG in males, and the change in serum RBP4 levels were related to change in FINS, FCP, HOMA-IR, and HOMA-β at 3rd month after LSG in females. Therefore, we assume that decreased RBP4 levels may contribute to improved glucose metabolism after LSG.

There also some limitations to our study. The sample size is relatively smaller, and the follow-up time is relatively short. We will further expand the sample size and extend the follow-up time to observe the change in RBP4 for the long-term.

## Conclusion

Overall, we relate the association between RBP4 with glucose metabolism, oxidative stress and thyroid function in patients of Chinese ethnicity. After the LSG surgery, the patients have shown improved glucose metabolism and decreased RBP4 levels in the 3rd and 6th months. Our study also suggests, decreased RBP4 levels may partly account for the improved glucose metabolism in obese patients after LSG. Future in-depth analyses on this area could project LSG as the most reliable treatment method for obesity and RBP4 as a trustable biomarker.

## Data Availability

Data are all contained within the paper.

## Abbreviations

RBP4: serum retinol-binding protein 4
LSG: laparoscopic sleeve gastrectomy
UA: uric acid
SOD: superoxide dismutase
FT3: free triiodothyronine
FT4: free thyroxin
TSH: thyrotropin
BMI: body mass index
FINS: fasting inulin
FCP: fasting C-peptide
HOMA-IR: homeostasis model of assessment for insulin resistance index
IFG: impaired fasting glucose
